# Production of Prebiotic Xylooligosaccharides via Dilute Maleic Acid-Mediated Xylan Hydrolysis Using an RSM-Model-Based Optimization Strategy

**DOI:** 10.3389/fnut.2022.909283

**Published:** 2022-05-10

**Authors:** Kankan Jiang, Xiaoliang Fu, Rong Huang, Xingli Fan, Lei Ji, Damin Cai, Xiaoxiang Liu, Yixiu Fu, Aihua Sun, Chenzhuo Feng

**Affiliations:** School of Basic Medical Sciences and Forensic Medicine, Hangzhou Medical College, Hangzhou, China

**Keywords:** Xylooligosaccharides, xylan, dilute maleic acid, hydrolysis, response surface methodology

## Abstract

Xylooligosaccharides (XOS) are functional feed additives that are attracting growing commercial interest owing to their excellent ability to modulate the composition of the gut microbiota. The acid hydrolysis-based processing of xylan-containing materials has been proposed to represent a cost-effective approach to XOS preparation, with organic acids being preferable in this context. As such, in the present study, maleic acid was selected as a mild, edible organic acid for use in the hydrolysis of xylan to produce XOS. A response surface methodology (RSM) approach with a central composite design was employed to optimize maleic acid-mediated XOS production, resulting in a yield of 50.3% following a 15 min treatment with 0.08% maleic acid at 168°C. Under these conditions, the desired XOS degree of polymerization (2-3) was successfully achieved, demonstrating the viability of this using a low acid dose and a high reaction temperature to expedite the production of desired functional products. Moreover, as maleic acid is a relatively stable carboxylic acid, it has the potential to be recycled. These results suggest that dilute maleic acid-based thermal treatment of corncob-derived xylan can achieve satisfactory XOS yields, highlighting a promising and cost-effective approach to XOS production.

## Introduction

Xylooligosaccharides (XOS) are non-digestible functional oligosaccharides with prebiotic activity, leading to their extensive use as food additives (1). As XOS exhibit excellent thermos-stability to 100°C and strong acid resistance across a pH range of 2.0–7.0, they are well-suited to gastrointestinal delivery given their ability to resist stomach acid (2). Several studies published to date have demonstrated that the use of XOS as a feed additive can improve specific growth rates and feed conversion ratio values in livestock while simultaneously promoting the growth of beneficial bacteria within the intestines and improving overall digestive performance and immune activity (3, 4). As a food additive, an intake of XOS (1–3 g/day) can readily promote the growth of probiotic bacterial species, enhance calcium absorption, protect against dental caries, and decrease the incidence of gastrointestinal and cardiovascular diseases (5, 6). Given their robust prebiotic properties, XOS have been a focus of growing scientific and commercial interest such that the market price for XOS is forecast to exceed $130 million by 2023 (2, 4).

Xylooligosaccharides are composed of 2–10 xylose units and are generated through the hydrolysis of xylan, which is the major extract from hemicelluloses composition of lignocellulosic biomass (7). XOS are currently produced by enzymatic method, chemical method, or a combination of these two approaches (2, 8). While enzymatic hydrolysis is more selective and can thus yield XOS of higher purity, it is expensive, slow, and necessitates specialized storage and handling of the utilized enzymes, thus making it poorly suited to large-scale commercial production (9, 10). In contrast, acid-based hydrolysis approaches are efficient and inexpensive such that they are more frequently employed in the context of large-scale industrial XOS production (11). The acidic hydrolysis techniques generally hydrolyze xylan using mineral acids or organic acids as catalysts (12–16). The use of conventional sulfuric acid, however, results in large quantities of inorganic waste in the form of gypsum together with significant quantities of other degradation products including pentose-derived furfural and hexose-derived 5-hydroxymethylfurfural. As organic acids yield fewer byproducts (xylose and furfural) in this reactive context, they are often favored over inorganic acids (17–20), with those byproducts produced during organic acid hydrolysis being more readily utilized in the form of combustion fuel in a co-firing device, as fertilizer, or as animal feed (19, 21, 22). Besides, simple soluble organic acids, e.g., mono-, di- and tri-carboxylic acids, are ubiquitous components of the nature system.

Maleic acid (MA) is a dibasic carboxylic acid that functions as an organic acid, releasing H^+^ to facilitate the depolymerization of xylan and other hemicelluloses, thereby yielding oligomers or monosaccharides. In prior reports, MA has been shown to be a safe and environmentally friendly organic acid that can be employed for the pretreatment of lignocellulosic materials. As the furfural byproduct derived from xylose dehydration necessitates higher activation energy, MA pretreatment can also lower byproduct yields as compared to sulfuric acid pretreatment (23). MA is also classified as a food additive, and it can thus be used in the co-preparation of XOS for use in feed additives. While MA has previously been reported in the context of monosaccharide preparation (23–26), few studies have explored its value in the context of XOS production. As such, in this study, MA was herein utilized to facilitate xylan hydrolysis as a means of generating XOS. Through single-factor and response surface methodology (RSM)-based approaches, MA-mediated XOS yields were optimized, and the interactive relationships between reaction temperature, acid content, and holding time were explored.

## Materials and Methods

### Materials

Corncobs that served as inputs in the present study were provided as a kind gift from farmers in Rizhao, Shandong province, China. Corncobs were initially milled and passed through 40–100 meshes, followed by use for xylan preparation. The prepared corncob was composed of 35.9% cellulose, 32.1% hemicellulose, and 17.3% lignin.

### Xylan Preparation

A two-step approach was used to prepare xylan through alkaline extraction and ethanol precipitation. Initially, the milled corncob was treated for 45 min with 7% NaOH at 120°C. Following alkaline treatment, the resultant sludge was centrifuged and the xylan-containing supernatant fraction was collected. Next, ice-cold 95% ethanol was used to precipitate these alkaline supernatants, with the precipitate then being collected and rinsed using 70% ethanol (10). Following this ethanol wash, precipitates were freeze-dried to facilitate downstream analyses and acidic hydrolysis. Xylan was assessed as per the standard method developed by the National Renewable Energy Laboratory's standard method (27).

### Maleic Acid-Mediated Xylan Hydrolysis

MA-mediated xylan hydrolysis was performed in a 100 mL screw-top pressure-resistant steel reactor. Briefly, 5 g of freeze-dried xylan powder and 50 mL of dilute MA prepared at the appropriate concentration were combined in the reactor at a 1:10 solid-liquid ratio. Xylan hydrolysis was performed using a constant temperature oil-bath pan (KEMAI, SC-10A, China). Reactors were sealed and immersed in an oil bath after stably reaching the selected preset temperature. After the selected reaction duration, reactors were removed and cooled in a cold water bath. The sludge therein was then centrifuged for 8 min at 8,000 rpm, after which supernatants were collected for analysis.

### Acid Hydrolysis Experimental Design

Experimental design was achieved using the response surface analysis software Design-Expert (v 11.0). For this analysis, three independent variables were selected, including reaction temperature (A, 130–170°C), acid concentration (B, 0.04%-0.16%, w/w), and hydrolysis time (C, 15–45 min). The XOS yield (**Y**) served as the response in this analysis, enabling the evaluation of interaction effects for reaction temperature, acid content, and holding time, with a quadratic polynomial then being generated via stepwise regression fitting. Significance for the designed experiments was assessed using analyses of variance (ANOVAs) (28). Model fit was assessed by calculating the *R*^2^ and adjusted *R*^2^ values, with model significance being established based on F- and *P*-values. Response surfaces were established with the fitted quadratic polynomial equation for each response as a means of demonstrating the effects of the selected variables on XOS yield.

### Analytical Methods

Xylose and XOS (including xylobiose (X2), xylotriose (X3), xylotetraose (X4), xylopentaose (X5), and xylohexaose (X6)) were simultaneously detected via High-performance anion-exchange chromatography (HPAEC) (Thermo Fisher, ICS-6000, USA) with a CarboPac™ PA200 column (29). MA and furfural were assessed via high performance liquid chromatography (HPLC) (Agilent 1200, USA), coupled with an Aminex Bio-Rad HPX-87H column (300 mm × 7.8 mm; USA) and a refractive index detector. An analytical column was operated at 55°C with a mobile phase consisting of 5 mM H_2_SO_4_. The flow rate for the mobile phase was 0.6 mL/min. Yields of xylose and XOS (X2-X6) were determined as follows:


(1) $$\begin{array}{l} Xylose\;yield\;\left(\%\right)=\frac{Xylose\;content\;in\;hydrolysate\;\left(g\right)}{The\;initial\;xylan\;content\;\left(g\right)}\times 100\% \end{array}$$

(2) $$\begin{array}{l} XOS\;yield\;\left(\%\right)=\frac{\left(X2+X3+X4+X5+X6\right)\;in\;hydrolysate\;\left(g\right)}{The\;initial\;xylan\;content\;\left(g\right)}\times 100\% \end{array}$$

## Results and Discussion

### Production of XOS in Single-Factor Experiments

**Figure 1:**
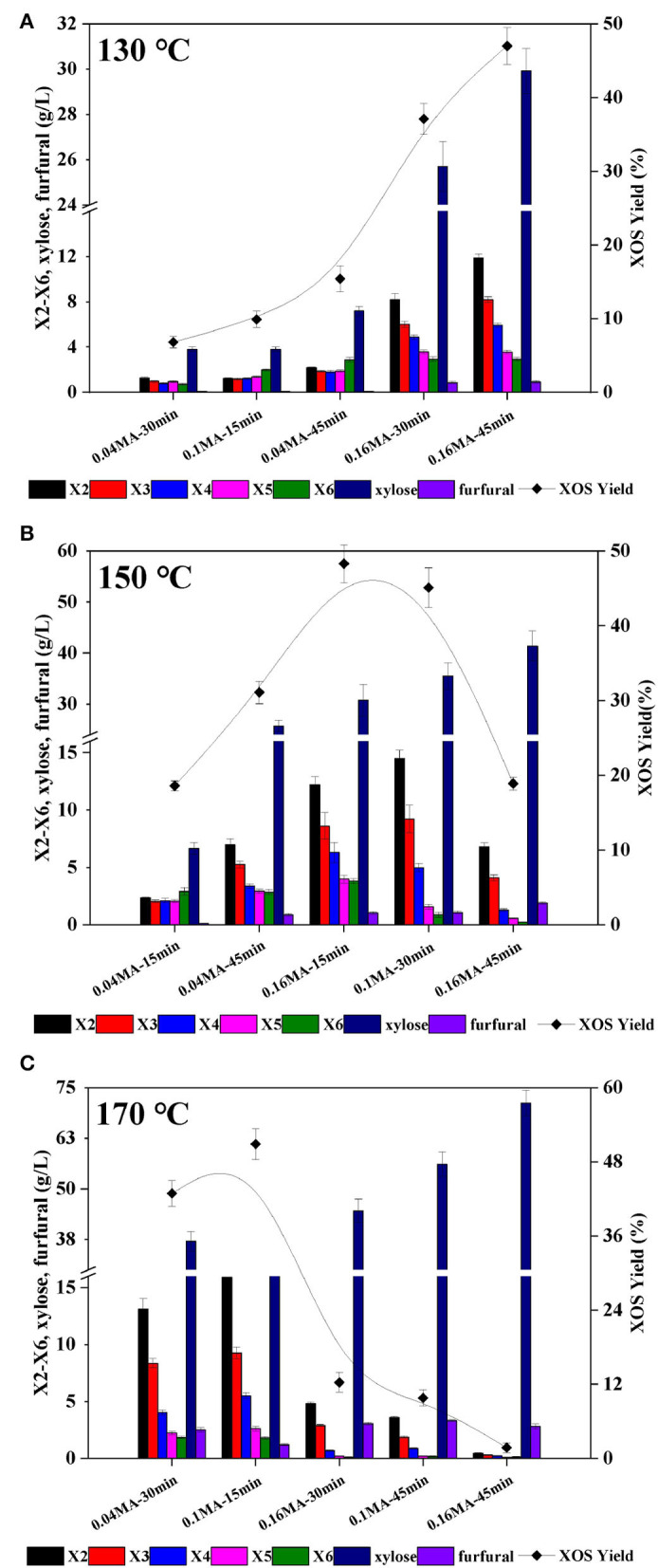
Furfural, xylose, and X2-X6 distributions and XOS yields in xylan hydrolysates prepared using a range of MA concentrations, holding times, and temperatures: **(A)** 130°C; **(B)** 150°C; and **(C)** 170°C. X2: xylobiose; X3: xylotriose; X4: xylotetraose; X5: xylopentaose; X6: xylohexaose; XOS: xylooligosaccharides = X2+X3+X4+X5+X6; MA: maleic acid.

Corncob extract-derived xylan was used for all XOS production in the present study. Briefly, alkaline corncob extracts from different batches were pooled to yield the experimental input material (69.1% xylan). The final recovery of xylan was 61.7% relative to the total xylan in raw material. Following alkaline treatment, the remaining non-xylan solids fraction was primarily comprised of cellulose, which can also serve as a raw source of glucan in the context of enzymatic hydrolysis-mediated glucose production (30). The rate of xylan degradation has previously been shown to be primarily influenced by reaction temperature, acid content, and holding time (11). An RSM experimental design approach was thus employed to explore the effects of these factors on XOS yields. In total, 15 individual independent experiments were performed at temperatures from 130–170°C (Figures 1A–C), with XOS yields (calculated based on the total X2–X6 content) and the distributions of xylose, furfural, and X2–X6 generated at a given fixed temperature being reported.

Under low reaction temperature and low acid content conditions, X2–X6 distributions were average, but total XOS yields were very low (<20%) (Figure 1A). For example, X2–X6 contents following treatment for 15 min at 130°C with 0.1% MA were 1.22, 1.16, 1.21, 1.36, and 1.59 g/L, respectively. When harsher reaction conditions were employed, however, declining trends in XOS concentrations were generally observed from X2–X6. At 130°C, XOS yields rose with increases in acid concentration or holding time. For example, following treatment at 130°C for 45 min with 0.04% MA, the XOS yield was just 10.6%, which was much lower than that observed under otherwise identical conditions with 0.16% MA (47.1%). At 150°C, the XOS yields rose with prolonged holding time at a low MA concentration (0.04%), but dropped sharply at holding times beyond 30 min when the acid content was >0.1% (Figure 1B). At 170°C, XOS yields declined with increases in acid content and holding time owing to the accelerated conversion of XOS to xylose under these conditions such that treatment for 45 min with 0.1% MA at 170°C for 45 min resulted in an XOS yield of just 9.8%, whereas the xylose yield was as high as 80.1% (Figure 1C). These data suggest that overly harsh reaction conditions can result in the excessive breakdown of xylan, making them ill-suited to preparing XOS (31). It is, however, important to note that furfural yields remained low (<5 g/L) under even the harshest conditions (170°C/45 min/0.16%), owing to the dicarboxylic structural characteristics of MA, which mimic the active sites of natural enzymes and thereby stabilize xylose, slowing its degradation (32). With appropriate acid concentrations and holding time values, high XOS yields of 45–50% were successfully achieved at 130, 150, and 170°C. While an XOS yield of 47.1% was achieved at 130°C (0.16% MA, 45 min), this required a notably prolonged reaction time, whereas at 150°C a 48.3% XOS yield was achieved in just 15 min using 0.16% MA. Overall, these experiments revealed that xylose production constantly increased with rising temperature, time, and MA concentration.

In acidic hydrolysis process, the glycosidic bonds between adjacent xylose units in xylan can be randomly hydrolyzed by hydronium ions, causing xylan depolymerization to give xylooligomers and xylose (13, 33, 34). Xylan is generally first broken down to yield polysaccharides with a higher degree of polymerization (DP) in acidic hydrolysis processes, followed by further degradation into oligomers with lower DP values (22). Under lower temperature conditions, higher XOS yields generally necessitate increases in either acid concentration or holding time in a manner that may be poorly suited to commercial applications. Increasing the holding time and acid concentrations can also result in further oligosaccharide degradation, yielding smaller byproducts including furfural and xylose. Reaction temperature, acid concentration, and holding time must therefore be synergistically regulated to improve XOS yields while minimizing the production of undesirable byproducts.

### Model Fitting

XOS yields were optimized using a 3-factor-3-level central composite design (CCD) strategy consisting of 15 experimental runs exploring relationships among reaction temperature (A), acid content (B), and holding time (C). For this approach, the central condition was as follows: 150°C, 0.1% MA, and 30 min. Each of the 15 runs was conducted in triplicate, with the resultant average XOS yields and corresponding ANOVAs being shown in Tables 1, 2, respectively.

**Table 1:** The experimental design and response value (XOS yield).

	**Factors**		**Response**
**A**	**B**	**C**	***Y***
Temperature /°C	Maleic acid /%	Holding time /min	XOS^a^ yield /%
130	0.04	30	6.8
	0.1	15	9.9
	0.04	45	10.6
	0.16	30	37.1
	0.16	45	47.1
150	0.04	15	18.6
	0.04	45	31.1
	0.16	15	48.3
	0.1	30	45.1
	0.16	45	18.9
170	0.04	30	42.9
	0.1	15	50.3
	0.16	30	12.3
	0.1	45	9.8
	0.16	45	1.7

a * XOS, xylooligosaccharides*.

**Table 2:** Analysis of variance in RSM model.

**Source**	**Sum of squares**	**Degree of freedom**	**Mean square**	***F*-value**	***P*-value**	
**Model**	3134.11	9	348.23	30.66	0.0085	significant
A-Temperature	71.81	1	71.81	6.32	0.0866	
B-Acid	55.11	1	55.11	4.85	0.1149	
C-Time	179.54	1	179.54	15.81	0.0285	
AB	1102.90	1	1102.90	97.10	0.0022	
AC	688.14	1	688.14	60.59	0.0044	
BC	456.67	1	456.67	40.21	0.0079	
A^2^	324.02	1	324.02	28.53	0.0128	
B^2^	196.52	1	196.52	17.30	0.0253	
C^2^	96.68	1	96.68	8.51	0.0616	
**Residual**	34.07	3	11.36			
**Cor Total**	3168.19	12				

A linear regression approach was successfully used to model XOS yields based on the above data, with the model *F*-value (30.66) corresponding to model significance given that there is only a 0.85% chance that such a value would only occur due to noise. Model terms with *P*-values < 0.05 and >0.1, respectively indicate significance and a lack of significance. In cases when many model terms not necessary to support the overall hierarchy of the model are insignificant, model reduction can lead to further improvements in the model. The signal-to-noise ratio (SNR) is a measure of precision, with an SNR >4 being desirable. Given the observed SNR of 16.388, the model signal was considered to be adequate such that it could be used to navigate the design space. A multiple regression analysis conducted with the Design-Expert software selected a quadratic model as being most appropriately matched to this model based on fit to the (**Y**). The resultant quadratic equation obtained through this analysis was as follows:

Y = 11.6A + 2950.7B + 9.8C−13.4AB-0.05AC−11.5BC−0.03A^2^-2753.5B^2^-0.03C^2^-1126.3, where A, B, and C, respectively denote temperature (°C), acid concentration (%), and holding time (min).

For this equation, positive and negative regression coefficient values correspond to synergistic effects and antagonistic effects, respectively. *F*-tests and *P*-values were used to assess the significance of each model term. As shown in Table 2, time significantly affected XOS yield (*P* < 0.05). Quadratic terms for acid concentration and temperature were also significant, which were the same as interactions between the two random factors.

**Figure 2:**
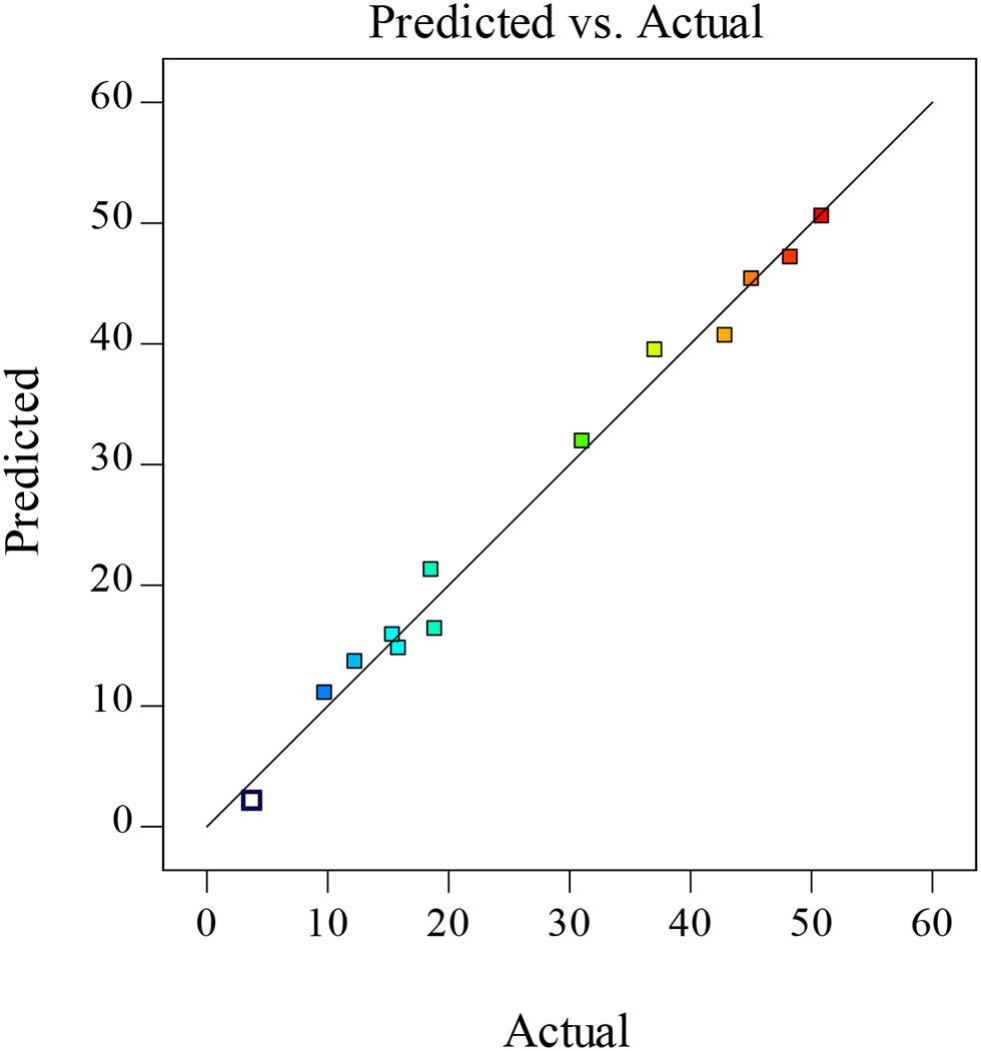
Correlations between predicted and actual values.

The coefficient (*R*^2^) and adjusted coefficient of determination (*Adj*.*R*^2^) values for this model were 0.98 and 0.93, respectively, with both being in good agreement and larger than 0.80, consistent with the fitted model exhibiting appropriate consistency between predicted and actual values (35). As the lack-of-fit value was not significant (not shown), this model was considered to be adequate as a tool for predictive analyses. In addition, strong linear correlations were visible between predicted and actual XOS yield values, confirming the reliability of this predictive model (Figure 2).

**Figure 3:**
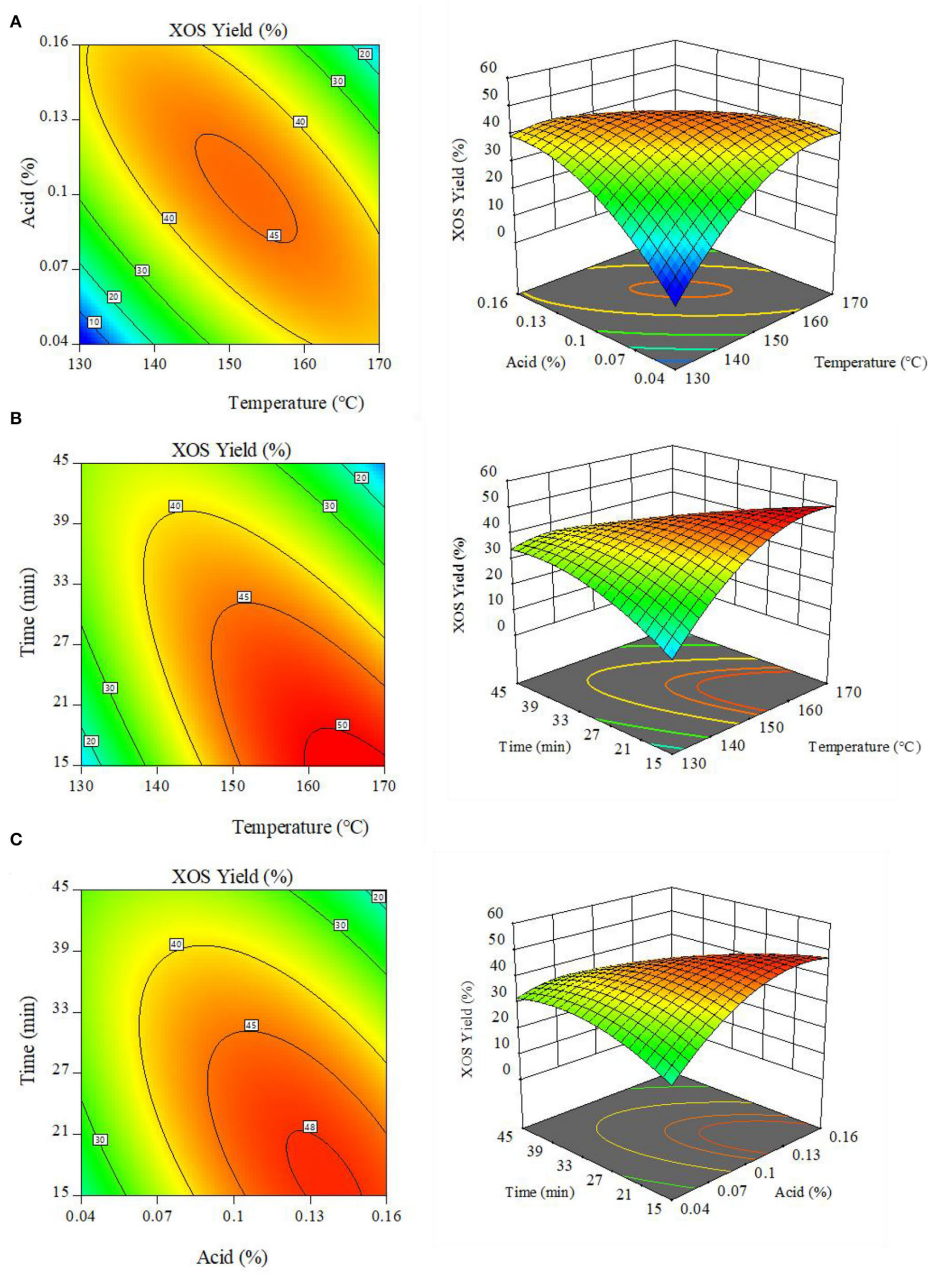
Response surface plots demonstrating the interactive effects of **(A)** reaction temperature and MA content. **(B)** reaction temperature and holding time; and **(C)** MA content and holding time on XOS production. XOS: xylooligosaccharides = X2+X3+X4+X5+X6.

The 3D response surface generated by the Design-Expert software enables the visualization of interactive effects associated with two factors when a third factor is fixed (Figures 3A–C). The observed results suggested that slight changes in reaction temperature, acid concentration, and holding time had the potential to significantly affect XOS yields. At a fixed 30 min holding time, the maximal contour with the greatest XOS yield (>45%) was obtained in the 145–160°C and 0.08–0.12% MA range (Figure 3A), with further increases in reaction temperature and acid concentration resulting in decreased XOS yields owing to the excessive conversion of XOS into xylose. Similar results were also observed when exploring the interaction effects of holding time and reaction temperature (Figure 3B) or holding time and acid concentration (Figure 3C).

### Predicted Value Verification

**Figure 4:**
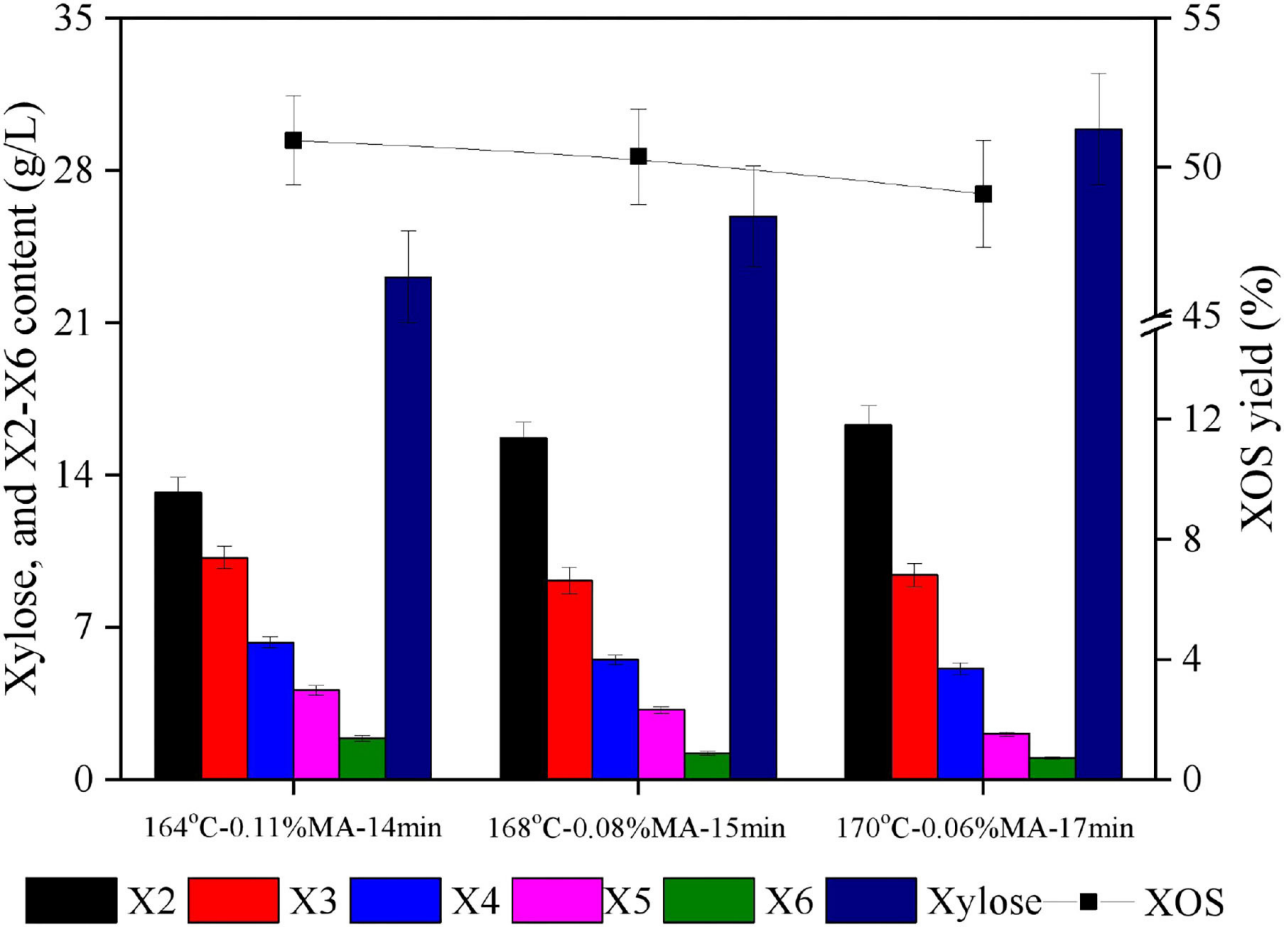
Hydrolysate component distributions and XOS yields for three chosen conditions. X2: xylobiose; X3: xylotriose; X4: xylotetraose; X5: xylopentaose; X6: xylohexaose; XOS: xylooligosaccharides = X2+X3+X4+X5+X6; MA: maleic acid.

In light of the 3D surface plots generated above, it was clear that holding time was the dominant factor significantly affecting XOS yield responses, as evidenced by a higher F-value for time relative to corresponding values for temperature or acid concentration (Table 2), with A, B, and C values in the 160–170°C, 0.08–0.12%, and 15–20 min ranges producing XOS yields of > 50%. In the fitted yield, the maximal predicted XOS yield (51.1%) was identified for the following reaction conditions: 164°C, 0.11% MA, and 14 min. From a practical industrial perspective, however, lower acid concentrations are preferable as they will lower the costs of downstream purification. Other response conditions with predicted XOS yields of around 50% were thus also compared (168°C, 0.08% MA, 15 min; 170°C, 0.06% MA, 17 min). Each of these experimental protocols was repeated in triplicate using the indicated reaction conditions, and xylan hydrolysis component distributions were then assessed (Figure 4). In the resultant hydrolysates, X2 and X3, which are considered to be the most effective prebiotic compounds (36), were the dominant components, while X4–X6 yields were substantially more limited.

The maximal achieved XOS yield under these experimental conditions was 51.7%, corresponding to a total concentration of 35.7 g/L. This yield was largely consistent with the predicted yield (51.1%) under the selected reaction conditions (164°C, 0.11% MA, 14 min), further supporting the accuracy of the established regression equation. XOS yields when xylan hydrolysis was performed using 0.06 or 0.08% MA were 49.1 and 50.3%, respectively. As these values were only slightly lower than the 51.7% yield achieved above, they were considered acceptable. At the 0.06% MA concentration, xylose byproduct levels were higher than those observed at a 0.08% MA concentration, leading to the conclusion that XOS production with 0.08% MA at 168°C for 15 min is preferable. This acid concentration is relatively low as compared to previously reported organic acid concentrations in other studies assessing xylan hydrolysis. For example, Zhang et al. (37) achieved a maximal 45.86% XOS yield when using 20% acetic acid to hydrolyze waste xylan extracted from viscose fiber plants for 20 min at 140°C. In another report, Zhao et al. (14) employed 1.2% furoic acid to facilitate the xylan hydrolysis of corncob xylan for 33 min at 167°C, achieving a 49.2% yield. Besides, it was reported that the highest XOS yield of 45.18% was obtained with 1% sulfuric acid at 120°C for 60 min during the hydrolysis of the residual hemicelluloses of dissolving pulp (38). Henriques et al. (39) concluded that a maximum XOS yield of 30.2% was obtained within 30 min at 100°C and pH 0.5 by nitric acid hydrolysis of xylan from *Eucalyptus globulus* bleached kraft pulp. The ability of dilute MA to efficiently hydrolyze xylan and generate XOS can be attributed to the low dissociation constant of MA (pKa1 = 1.83, pKa2 = 6.07) (40, 41). This property, in turn, is attributable to the fact that MA is monounsaturated and protonates via the formation of a stable cis-anion through intramolecular hydrogen bonding. The sp^2^ character of MA further improves its acidity as its electrons are more closely located to the unsaturated carbon nucleus, resulting in a lower energy state and enhanced anion stability (42). MA is also free of additional alkyl groups that can inductively decrease acid strength. Together, these features are conducive to the enhanced acidity of MA such that it is more similar to a strong acid capable of readily releasing H^+^ (43). The activation energy for MA-mediated hemicellulose hydrolysis is significantly lower than that for sulfuric acid, suggesting MA to be a more efficient catalyst in this context (44). Other studies have also reported MA to exhibit greater efficacy when converting barley straw xylan into reducing sugar as compared to other monocarboxylic or dicarboxylic acids, including adipic acid, benzoic acid, malonic acid, and salicylic acid (42). Lin et al. (11) have also further reported MA to be better suited than citric acid to the catalysis of hemicellulose conversion into XOS.

**Figure 5:**
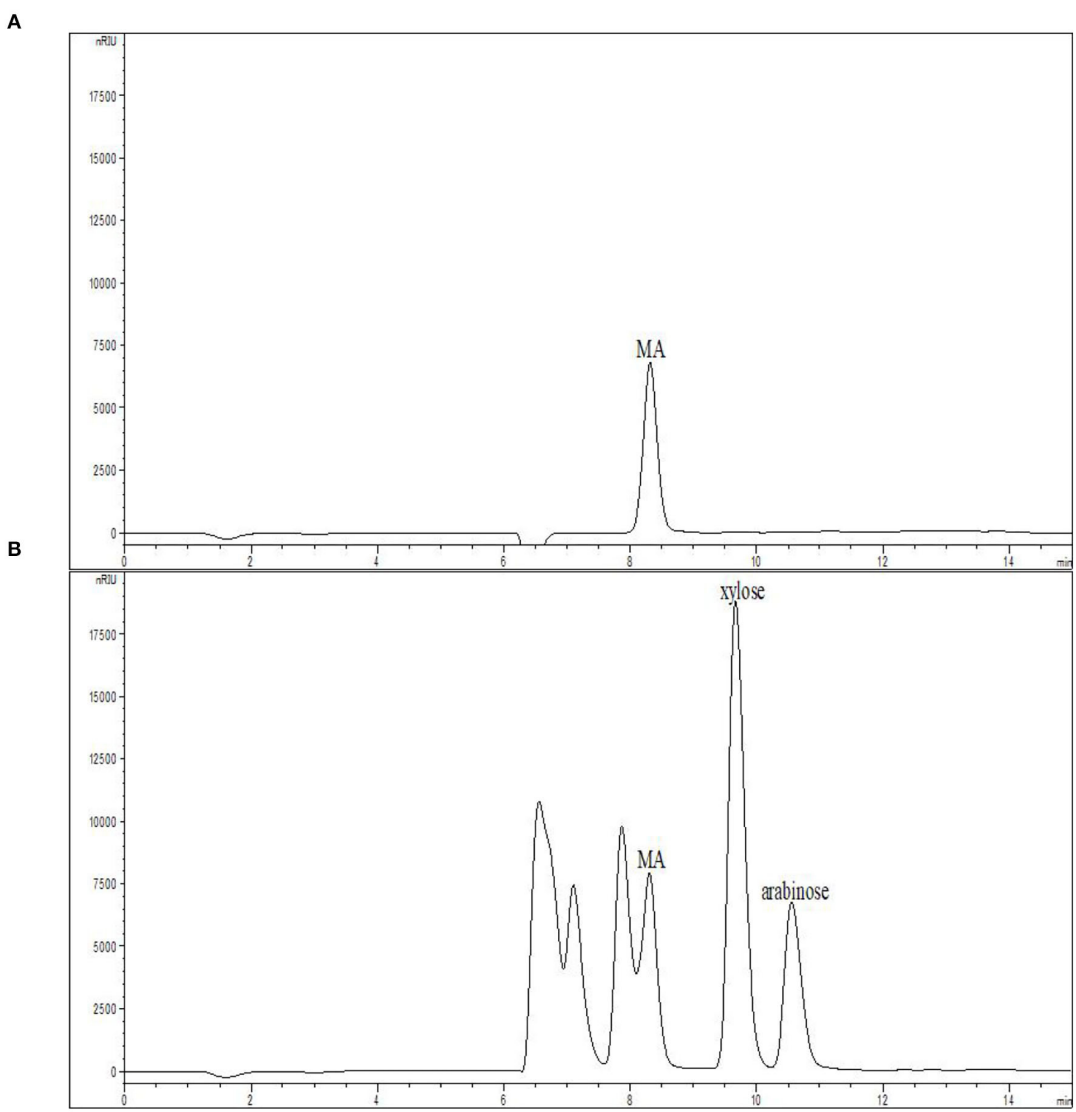
MA solution chromatograms **(A)** prior to and **(B)** following xylan hydrolysis at 168°C with 0.08% MA for 15 min. MA, maleic acid.

The feasible commercial use of MA as a catalyst necessitates that it can be repeatedly recycled. MA chromatograms before and after the hydrolysis of xylan at 168°C with 0.08% MA for 15 min were compared in Figure 5. While partial overlap of the MA and XOS peaks was observed, the intensity of the post-hydrolysis MA peak was very similar to the pre-hydrolysis peak, suggesting that MA does not undergo degradation or conversion during hydrolysis. As such, MA offers great potential for the industrial-scale production of XOS given that it can potentially be recovered via electro-dialysis procedures (45).

## Conclusions

In summary, dilute edible MA was employed to produce XOS via an acid hydrolysis approach using xylan as an input, with an RSM approach being used to optimize the necessary reaction temperature, holding time, and acid concentration. Using this strategy and quadratic regression model-based predictions, an optimal 50.3% XOS yield was achieved, highlighting a promising approach to achieving large-scale production of XOS with desired DP (X2 and X3).

## Data Availability Statement

The raw data supporting the conclusions of this article will be made available by the authors, without undue reservation.

## Author Contributions

CF and AS developed the idea and methodology for the study and helped in manuscript preparation. KJ and XFu performed the experiment and drafted the manuscript. RH, XFa, LJ, DC, XL, and YF analyzed the results and interpreted them. All authors contributed to manuscript revision, read, and approved the submitted version.

## Funding

The works were supported by Scientific Research Fund of Zhejiang Provincial Education Department (Y202146043), the Scientific Research Fund of Zhejiang Provincial Education Department (Y202146041), Basic Research Funds of Hangzhou Medical College (KYYB202009), Doctoral Scientific Research Foundation of Hangzhou Medical College (00004F1RCYJ2001), Zhejiang Provincial Natural Science Foundation of China (No. LY20C200002), Basic Research Funds of Hangzhou Medical College (KYQN202104), and Zhejiang Provincial Program for the Cultivation of High-Level Innovative Health Talents.

## Conflict of Interest

The authors declare that the research was conducted in the absence of any commercial or financial relationships that could be construed as a potential conflict of interest.

## Publisher's Note

All claims expressed in this article are solely those of the authors and do not necessarily represent those of their affiliated organizations, or those of the publisher, the editors and the reviewers. Any product that may be evaluated in this article, or claim that may be made by its manufacturer, is not guaranteed or endorsed by the publisher.
